# Supplementary material for: Suitability of paddy cultivation in the Western province of Sri Lanka under different climate change scenarios
Source: PLoS One. 2025 Oct 27;20(10):e0333100. doi: 10.1371/journal.pone.0333100 (PMC12558550; doi:10.1371/journal.pone.0333100)
Supplement: Attachment — Submitted filename: Response to Reviewers.docx [file pone.0333100.s002.docx]

To:

Academic Editor and Reviewer,

PLOS ONE.

27/06/2025

Subject: Addressing Academic Editor’s and Reviewer’s Comments for PONE-D-25-10830

Dear academic editor and reviewer,

We thank you for the time and effort invested in evaluating our manuscript, titled “Suitability of paddy cultivation in the Western province of Sri Lanka under different climate change scenarios”. We sincerely appreciate the thoughtful and constructive feedback, which has greatly improved the quality of our manuscript.

Below, we provide a point-by-point response to each of the comments. Our response for each comment is highlighted in **red**.

1. **Comments by the academic editor**

Please include the following items when submitting your revised manuscript:

- A rebuttal letter that responds to each point raised by the academic editor and reviewer(s). You should upload this letter as a separate file labeled 'Response to Reviewers'.
- Included.
- A marked-up copy of your manuscript that highlights changes made to the original version. You should upload this as a separate file labeled 'Revised Manuscript with Track Changes'.
  - Included.
- An unmarked version of your revised paper without tracked changes. You should upload this as a separate file labeled 'Manuscript'.
  - Included.

If you would like to make changes to your financial disclosure, please include your updated statement in your cover letter. Guidelines for resubmitting your figure files are available below the reviewer comments at the end of this letter.

- Changes to the financial disclosure statement have been updated in the cover letter.

If applicable, we recommend that you deposit your laboratory protocols in protocols.io to enhance the reproducibility of your results. Protocols.io assigns your protocol its own identifier (DOI) so that it can be cited independently in the future. For instructions see: <https://journals.plos.org/plosone/s/submission-guidelines#loc-laboratory-protocols>. Additionally, PLOS ONE offers an option for publishing peer-reviewed Lab Protocol articles, which describe protocols hosted on protocols.io. Read more information on sharing protocols at <https://plos.org/protocols?utm_medium=editorial-email&utm_source=authorletters&utm_campaign=protocols>.

- Not applicable.

2. **Journal Requirements**

When submitting your revision, we need you to address these additional requirements.

1. Please ensure that your manuscript meets PLOS ONE's style requirements, including those for file naming. The PLOS ONE style templates can be found at

https://journals.plos.org/plosone/s/file?id=wjVg/PLOSOne_formatting_sample_main_body.pdf and

<https://journals.plos.org/plosone/s/file?id=ba62/PLOSOne_formatting_sample_title_authors_affiliations.pdf>.

- We have now followed PLOS ONE’s style requirements, including those for file naming.
- A corresponding author has been added to the title page, and it is in accordance with PLOS ONE’s style requirements.
- An ‘Acknowledgments’ section has been added to recognize contributions to GIS support and map development, and it is in accordance with PLOS ONE’s style requirements.

2. In your Methods section, please provide additional information regarding the permits you obtained for the work. Please ensure you have included the full name of the authority that approved the field site access and, if no permits were required, a brief statement explaining why.

- The following statement has been included in the manuscript under the Methods section; “This research did not involve collection of data from the public as the method was mainly involved in modelling, model application, model validation, model calibration and model simulation. The research proposal was presented to the Department of Zoology and Environmental Management, Faculty of Science, University of Kelaniya, and the proposal and the methodology were approved. As the research did not involve any data collection from any community or public, ethical review or approval was not necessary. Further, the field verification on the availability of paddy fields was done based on satellite images. The field verification and primary data collection of paddy fields were done by visiting the locality for which no approval is needed from any authority under the local context as they are openly accessible to the public.”

3. Please note that PLOS ONE has specific guidelines on code sharing for submissions in which author-generated code underpins the findings in the manuscript. In these cases, we expect all author-generated code to be made available without restrictions upon publication of the work. Please review our guidelines at https://journals.plos.org/plosone/s/materials-and-software-sharing#loc-sharing-code and ensure that your code is shared in a way that follows best practice and facilitates reproducibility and reuse.

- Not applicable.

4. Please include a complete copy of PLOS’ questionnaire on inclusivity in global research in your revised manuscript. Our policy for research in this area aims to improve transparency in the reporting of research performed outside of researchers’ own country or community. The policy applies to researchers who have travelled to a different country to conduct research, research with Indigenous populations or their lands, and research on cultural artefacts. The questionnaire can also be requested at the journal’s discretion for any other submissions, even if these conditions are not met.  Please find more information on the policy and a link to download a blank copy of the questionnaire here: https://journals.plos.org/plosone/s/best-practices-in-research-reporting. Please upload a completed version of your questionnaire as Supporting Information when you resubmit your manuscript.

- A complete copy of PLOS’ questionnaire on inclusivity in global research has been included as Supporting Information when submitting the revised manuscript.
- A reference to the checklist has been made in the main body of the manuscript by adding the subsection ‘Inclusivity in global research’ to the Methods section and adding the following sentence: “Additional information regarding the ethical, cultural, and scientific considerations specific to inclusivity in global research is included in the Supporting Information (S1 Checklist).”

5. Thank you for stating the following financial disclosure:

 [This research was supported by Researchers Supporting Project number (RSP2024R443), King Saud University, Riyadh, Saudi Arabia.].

Please state what role the funders took in the study.  If the funders had no role, please state: ""The funders had no role in study design, data collection and analysis, decision to publish, or preparation of the manuscript.""

If this statement is not correct you must amend it as needed.

Please include this amended Role of Funder statement in your cover letter; we will change the online submission form on your behalf.

- The above-mentioned statement relating to the financial disclosure has been amended in the cover letter.
- The amended Role of the Funder statement has been included in the cover letter.

6. We note that your Data Availability Statement is currently as follows: [All relevant data are within the manuscript.]

Please confirm at this time whether or not your submission contains all raw data required to replicate the results of your study. Authors must share the “minimal data set” for their submission. PLOS defines the minimal data set to consist of the data required to replicate all study findings reported in the article, as well as related metadata and methods (https://journals.plos.org/plosone/s/data-availability#loc-minimal-data-set-definition).

For example, authors should submit the following data:

- The values behind the means, standard deviations and other measures reported;

- The values used to build graphs;

- The points extracted from images for analysis.

Authors do not need to submit their entire data set if only a portion of the data was used in the reported study.

If your submission does not contain these data, please either upload them as Supporting Information files or deposit them to a stable, public repository and provide us with the relevant URLs, DOIs, or accession numbers. For a list of recommended repositories, please see https://journals.plos.org/plosone/s/recommended-repositories.

If there are ethical or legal restrictions on sharing a de-identified data set, please explain them in detail (e.g., data contain potentially sensitive information, data are owned by a third-party organization, etc.) and who has imposed them (e.g., an ethics committee). Please also provide contact information for a data access committee, ethics committee, or other institutional body to which data requests may be sent. If data are owned by a third party, please indicate how others may request data access.

- We confirm that our submission contains all raw data required to replicate the results of our study.

6. Please review your reference list to ensure that it is complete and correct. If you have cited papers that have been retracted, please include the rationale for doing so in the manuscript text, or remove these references and replace them with relevant current references. Any changes to the reference list should be mentioned in the rebuttal letter that accompanies your revised manuscript. If you need to cite a retracted article, indicate the article’s retracted status in the References list and also include a citation and full reference for the retraction notice.

- We have reviewed and reformatted the reference list in compliance with PLOS ONE guidelines (author names, title casing, journal presentation, DOIs). We confirm that none of the citations have been retracted. Reference formatting changes are highlighted in the revised manuscript. New references (45, 46, 48, 50 and 51) have been added to the revised manuscript.

3. **Comments by the Reviewer**

1. Is the manuscript technically sound, and do the data support the conclusions?

The manuscript must describe a technically sound piece of scientific research with data that supports the conclusions. Experiments must have been conducted rigorously, with appropriate controls, replication, and sample sizes. The conclusions must be drawn appropriately based on the data presented.

Reviewer #1: Yes

________________________________________

2. Has the statistical analysis been performed appropriately and rigorously?

Reviewer #1: Yes

________________________________________

3. Have the authors made all data underlying the findings in their manuscript fully available?

The PLOS Data policy requires authors to make all data underlying the findings described in their manuscript fully available without restriction, with rare exception (please refer to the Data Availability Statement in the manuscript PDF file). The data should be provided as part of the manuscript or its supporting information, or deposited to a public repository. For example, in addition to summary statistics, the data points behind means, medians and variance measures should be available. If there are restrictions on publicly sharing data—e.g. participant privacy or use of data from a third party—those must be specified.

Reviewer #1: Yes

________________________________________

4. Is the manuscript presented in an intelligible fashion and written in standard English?

PLOS ONE does not copyedit accepted manuscripts, so the language in submitted articles must be clear, correct, and unambiguous. Any typographical or grammatical errors should be corrected at revision, so please note any specific errors here.

Reviewer #1: Yes

________________________________________

5. Review Comments to the Author

Please use the space provided to explain your answers to the questions above. You may also include additional comments for the author, including concerns about dual publication, research ethics, or publication ethics. (Please upload your review as an attachment if it exceeds 20,000 characters)

Reviewer #1: The manuscript titled "Assessment of Paddy Distribution in the Western Province Under Different Climate Change Projections" presents a relevant and timely study. The research topic is well-conceived and holds significant potential for both scientific understanding and practical applications in agriculture and climate adaptation. However, the manuscript would benefit from several improvements in both content presentation and scientific rigor.

• The manuscript requires a thorough revision for English language usage. Several grammatical errors and stylistic inconsistencies currently hinder readability. Consider professional language editing to enhance clarity and flow.

- The English language usage of the manuscript was revised through professional language editing

• The Introduction needs sharpening to clearly articulate the study’s motivation, research questions, and knowledge gaps.

- The introduction was amended to include the study’s motivation, research questions, and knowledge gaps.

• The captions for all tables and figures need substantial revision. They should be self-explanatory and include enough detail to understand the content without referring back to the main text.

- The captions of all tables and figures were revised accordingly.

• In particular, Tables 1, 2, and 3 lack descriptive titles and context. Please include information on variable definitions, data sources, and any abbreviations used.

- The titles of Tables 1, 2 and 3 (Tables 2, 3 and 4 in the revised manuscript) were revised accordingly. Legend, footnotes have been added to include information on variable definitions, data sources, and any abbreviations used.

• The manuscript lacks clear identification of the climate models used for current and future projections section in Result. Please specify the model names selected for the assessment.

- The climate models used for current and future projections have been specified in the Results section, specifically the section relating to the ‘Individual model accuracy’.

• The discussion of predictor variable importance is repeated in both the methodology and results sections. This redundancy should be addressed.

- This redundancy has been addressed by removing the discussion of predictor variable importance from the Results section.

• Include a dedicated figure or table summarizing variable importance rankings, and quantify the relative contribution of each predictor to the model outcome.

- Figure 4 and Table 1 were included to summarize the variable importance rankings and the relative contribution of each predictor to the model outcome, respectively.

• Several Maps lack sufficient detail. Coordinate values are either too small or not visible. Please improve the clarity of all spatial maps by enhancing font size, scale bar visibility, and legend formatting.

- The clarity of all the maps has been improved.

• It is recommended to include an elevation profile within the study area map of the Western Province to provide additional geographical context relevant to paddy distribution.

- An elevation profile within the study area map of the Western Province has been included as Figure 1.

• The rice occurrence points would be more informative if overlaid on a true-color (RGB) satellite composite. This would enhance the visual contrast between land cover types and highlight agricultural patterns.

- The rice occurrence points have been overlaid on a true-colour (RGB) satellite composite (Figure 2).

• Figures 2 and 3 appear to present overlapping data. Consider combining them into a single comparative figure to streamline the presentation and reduce redundancy.

- While Figures 2 and 3 share overlapping data regarding the presence of paddy fields, we have retained Figure 3 because it was generated using the biomod2 package to specifically highlight the areas where paddy fields are absent. This distinction provides valuable information for comparative analysis, which would be lost if the figures were combined. Therefore, we respectfully request to keep both figures to preserve the completeness and clarity of the results.

• Figures 4, 5, and 7 do not mention the specific models used and replace generic identifiers like “Model 1” with the actual model names for transparency.

- The models used for Figures 4, 5 and 7 (Figures 5 and 7 in the revised manuscript) have been specified by amending the respective Figure captions.

• To enable comparative analysis, it would be beneficial to consolidate outputs from all climate models into a single composite figure. This would allow readers to visually assess inter-model variation in predicted paddy distribution.

- The outputs from all climate models have been consolidated into a single composite figure (Figure 5).

• The Discussion currently lacks focus. Strengthen it by directly comparing results with past studies, highlighting implications, limitations, and directions for future research.

- The Discussion has been amended accordingly.

**________________________________________**

6. PLOS authors have the option to publish the peer review history of their article (what does this mean?). If published, this will include your full peer review and any attached files.

If you choose “no”, your identity will remain anonymous but your review may still be made public.

Do you want your identity to be public for this peer review? For information about this choice, including consent withdrawal, please see our Privacy Policy.

Reviewer #1: No

________________________________________

[NOTE: If reviewer comments were submitted as an attachment file, they will be attached to this email and accessible via the submission site. Please log into your account, locate the manuscript record, and check for the action link "View Attachments". If this link does not appear, there are no attachment files.]

While revising your submission, please upload your figure files to the Preflight Analysis and Conversion Engine (PACE) digital diagnostic tool, https://pacev2.apexcovantage.com/. PACE helps ensure that figures meet PLOS requirements. To use PACE, you must first register as a user. Registration is free. Then, login and navigate to the UPLOAD tab, where you will find detailed instructions on how to use the tool. If you encounter any issues or have any questions when using PACE, please email PLOS at figures@plos.org. Please note that Supporting Information files do not need this step.

- The figure files were uploaded to the Preflight Analysis and Conversion Engine (PACE) digital diagnostic tool. Therefore, we confirm that the figures meet PLOS ONE requirements.

________________________________________

In compliance with data protection regulations, you may request that we remove your personal registration details at any time. (Remove my information/details). Please contact the publication office if you have any questions.

We thank the reviewers and the editorial team for the opportunity to revise our manuscript. We hope that the revisions address the concerns raised and that the improved manuscript is now suitable for publication in PLOS ONE.

Sincerely,

On behalf of all authors
Liyana Mudiyanselage Kasuni Gayathree Pitawala,
Department of Zoology and Environmental Management, Faculty of Science, University of Kelaniya, Sri Lanka.
[kasuni.pitawala@gmail.com](mailto:kasuni.pitawala@gmail.com)
